# Insomnia symptoms as a cause of type 2 diabetes Incidence: a 20 year cohort study

**DOI:** 10.1186/s12888-017-1268-4

**Published:** 2017-03-16

**Authors:** Michael J Green, Colin A Espie, Frank Popham, Tony Robertson, Michaela Benzeval

**Affiliations:** 1 MRC/CSO Social & Public Health Sciences Unit, University of Glasgow, 200 Renfield Street, Glasgow, G2 3QB UK; 2 Nuffield Department of Clinical Neurosciences, Sleep & Circadian Neuroscience Institute, University of Oxford, Oxford, OX3 9DU UK; 3 Faculty of Health Sciences and Sport, University of Stirling, Stirling, FK9 4LA UK; 4 Institute for Social and Economic Research, University of Essex, Colchester, CO4 3SQ UK; 5 Institute of Health and Wellbeing, University of Glasgow, 1 Lilybank Gardens, Glasgow, G12 8RZ UK

**Keywords:** Insomnia, Sleep, Type 2 Diabetes, Longitudinal, Confounding, Causal Effects, Marginal Structural Models

## Abstract

**Background:**

Insomnia symptoms are associated with type 2 diabetes incidence but are also associated with a range of potential time-varying covariates which may confound and/or mediate associations. We aimed to assess whether cumulative exposure to insomnia symptoms has a causal effect on type 2 diabetes incidence.

**Methods:**

A prospective cohort study in the West of Scotland, following respondents for 20 years from age 36. 996 respondents were free of diabetes at baseline and had valid data from up to four follow-up visits. Type 2 diabetes was assessed at the final visit by self-report, taking diabetic medication, or blood-test (HbA_1c_ ≥ 6.5% or 48 mmol/mol). Effects of cumulative insomnia exposure on type 2 diabetes incidence were estimated with traditional regression and marginal structural models, adjusting for time-dependent confounding (smoking, diet, physical inactivity, obesity, heavy drinking, psychiatric distress) as well as for gender and baseline occupational class.

**Results:**

Traditional regression yielded an odds ratio (OR) of 1.34 (95% CI: 1.06-1.70) for type 2 diabetes incidence for each additional survey wave in which insomnia was reported. Marginal structural models adjusted for prior covariates (assuming concurrently measured covariates were potential mediators), reduced this OR to 1.20 (95% CI: 0.98-1.46), and when concurrent covariates were also included (viewing them as potential confounders) this dropped further to 1.08 (95% CI: 0.85-1.37).

**Conclusions:**

The association between cumulative experience of insomnia and type 2 diabetes incidence appeared confounded. Evidence for a residual causal effect depended on assumptions as to whether concurrently measured covariates were confounders or mediators.

**Electronic supplementary material:**

The online version of this article (doi:10.1186/s12888-017-1268-4) contains supplementary material, which is available to authorized users.

## Background

Insomnia symptoms (defined here as trouble initiating or maintaining sleep) are associated with risk of type 2 diabetes [1–3]. Insomnia can be effectively treated [4] and may be a promising avenue for interventions to reduce type 2 diabetes incidence, as there are plausible biological mechanisms linking sleep loss to the development of type 2 diabetes via increases in insulin resistance and appetite [5, 6].

However, treatment of insomnia will only be effective at reducing risk of type 2 diabetes if the relationship between insomnia symptoms and type 2 diabetes is causal. Whilst observational studies show associations between insomnia symptoms and type 2 diabetes incidence [1–3], these may be confounded by other mechanisms. Indeed, socioeconomic position (SEP), smoking, heavy drinking, physical inactivity, poor diet and psychiatric distress are all associated with increased risk of insomnia symptoms or short sleep duration as well as type 2 diabetes [7–19]. Psychiatric distress and being overweight/obese may even have reciprocal relationships with sleeping trouble where each aggravates the other [6, 19]. This paper aims to assess whether associations between insomnia symptoms and type 2 diabetes incidence are likely to be causal by using marginal structural models (MSMs) to effectively control for time-varying confounders, and comparing results with more traditional regression models.

**Fig. 1:**
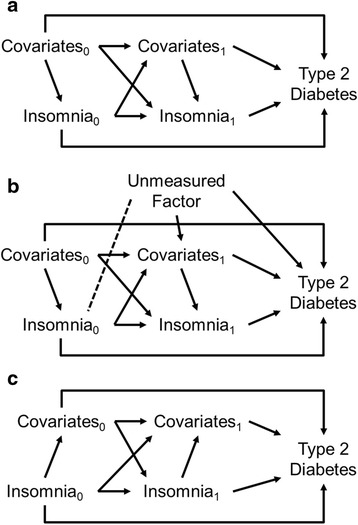
Causal diagrams of time-varying confounding **a** time-varying confounding with covariates influencing concurrent insomnia; **b** collider bias that can occur when conditioning on time-varying covariates; **c** time-varying confounding with covariates influenced by concurrent insomnia

Consider Fig. 1a, where time-varying insomnia symptoms are both influenced by, and influence, a set of time-varying covariates (e.g. smoking, physical inactivity etc), and both insomnia and these covariates are determinants of type 2 diabetes (the subscripts 0 and 1 indicate that insomnia and the covariates are measured at two successive time-points). The time-varying covariates may both confound associations between insomnia and type 2 diabetes (e.g. insomnia_1_ to type 2 diabetes is confounded by covariates_0_ and covariates_1_), but may also mediate causal effects of insomnia on type 2 diabetes (i.e. insomnia_0_ to covariates_1_ to type 2 diabetes). Mediation might occur for example if poor sleep increased appetite leading to poor diet/obesity and then to type 2 diabetes.

Assuming no further unmeasured confounding of the relationship between insomnia and type 2 diabetes, traditional regression approaches which condition on time-varying covariates will give biased estimates of the causal effect of insomnia. There are two reasons for this. First, conditioning on time-varying covariates simultaneously removes both their confounding influence and their mediated effect [20]. Confounding influences should be removed from effect estimates but mediated effects should not. Second, if some unmeasured factor confounds the association between the time-varying covariates and type 2 diabetes (as in Fig. 1b), conditioning on the time-varying covariates induces an association between that unmeasured factor and insomnia (represented by the dashed line), opening up a path from insomnia to type 2 diabetes which does not run via the time-varying covariates and is not causal, thus biasing estimates of insomnia’s effect. This is known as collider bias [21]. Both problems are still issues where insomnia and covariates are only measured at a single time-point (e.g. if only insomnia_1_ and covariates_1_ were observed) as the relevant mediating pathways may still be present, just unobserved (indeed, in this instance covariates_0_ could be the unmeasured confounder introducing collider bias).

MSMs avoid both of these problems by using the covariates to calculate analysis weights rather than by conditioning on them [20, 22]. The weights create a pseudo-population where at each time point those with and without insomnia will have similar covariate histories, but might differ systematically in covariates measured after that time point. This allows for mediation via covariates and does not induce the dashed association which causes collider bias in Fig. 1b. This paper compares traditional regression analysis with MSMs to explore the impact of effectively adjusting for time-varying covariates on our understanding of the causal relationship between insomnia and diabetes.

We additionally explore whether conclusions are robust to different causal assumptions. Where insomnia symptoms and time-varying covariates are measured concurrently, Fig. 1a and b show the conservative assumption that causation runs from the covariates to insomnia. Under this assumption concurrently measured covariates should be included when calculating weights for MSMs. An alternative, less conservative, assumption is depicted in Fig. 1c. In this case, concurrently measured covariates are viewed as caused by insomnia, and hence as mediators of insomnia’s effect. This means concurrent covariates should not be included when calculating weights, while earlier measurements of covariates are still potential confounders and should be included. We repeat analyses with and without concurrent covariates included in the weighting to see if conclusions are sensitive to these assumptions.

## Methods

### Sample

Data are from the Twenty-07 Study [23] which followed people in three age cohorts – born around 1932, 1952, and 1972 - for 20 years. It has two samples: the regional sample, a two-stage stratified random sample of people living in an area of the West of Scotland centred on Glasgow (previously known as the Central Clydeside Conurbation), and the localities sample of people from two areas of the city of Glasgow. Baseline interviews were conducted in 1987/88 and there have been four waves of follow-up (1990/2; 1995/7; 2000/4; 2007/8). This paper focuses on the 1950s birth cohort who aged from approximately 36 to 57 years during the study period, covering the age range in which type 2 diabetes is most likely to develop. This cohort had a baseline sample size of 1444 (the response rate was 88.9%), which has been shown to be representative of the general population of the sampled area [24]. The analysis sample for this paper was a sub-set of respondents from the 1950s cohort who participated in both the baseline and the final interview and were free of diabetes at baseline (*n* = 996; 73% of the living baseline sample; *n* = 3 excluded for baseline diabetes; *n* = 445 did not participate at the final interview). Implications of sample attrition are discussed below.

### Measures

Type 2 diabetes was indicated by either self-reporting of the condition, self-reporting of diabetic medication (medications coded 6.1 in the British National Formulary) [25], or blood-test results indicating HbA_1c_ ≥ 6.5% (48 mmol/mol). Respondents without valid blood results (*n* = 125) were still included and coded based on self-reported conditions and medications. Overall 68 respondents were coded as developing type 2 diabetes (28 by self-reported conditions or medications only, 10 by blood test results only, and 30 by both self-report and blood test). Baseline exclusions of existing cases were made on the basis of self-reported conditions/medications only as blood tests were not available until the final interview. A sensitivity analysis used a less conservative cut off of HbA_1c_ ≥ 6% (42 mmol/mol; identifying a further 50 potential cases).

Gender was coded 0 for males and 1 for females. SEP was measured as baseline household occupational class, coded according to the Registrar General’s 1980 classification [26], using the higher status occupation from couple households, with a binary indicator for manual vs non-manual occupations. This well-validated measure represents the material resources available to the household as well as their social standing [27].

In the first three interviews respondents were asked “How often do you have trouble getting to sleep?” and “How often are you bothered by waking earlier than you would like to, or by waking up in the middle of the night?” Both questions had six available responses ranging from never to every day. In the fourth and fifth interviews, respondents were asked, as part of the Pittsburgh Sleep Quality Index [28], “During the past month how often have you had trouble sleeping because you cannot get to sleep within 30 minutes?” and “During the past month how often have you had trouble sleeping because you wake up in the middle of the night or early morning?” Both questions had four response categories ranging from not during the past month to 3 or more times a week. Responses indicating at least weekly trouble initiating or maintaining sleep were coded as insomnia symptoms (a sensitivity analysis where insomnia was defined as weekly trouble with both initiation and maintenance of sleep produced similar results).

Current smoking, physical activity, past week alcohol consumption and fruit and vegetable consumption (as an indication of diet) were self-reported at each wave. For consistency across all confounders, they were coded as binary indicators. Respondents who reported any current smoking were coded as smokers. Physical inactivity was coded as not taking part in any weekly activity “lasting more than 20 min” that made them “sweat or out of breath”. Heavy drinking, based on past week alcohol consumption, was coded as >14 units for women and >21 units for men [29]. Less than daily consumption of fruit and vegetables was coded as poor diet. Height (m) and weight (kg) were recorded by trained nurses and used to calculate body mass index (BMI) at each wave (weight/height^2^). A BMI of 30+ was coded as obese (similar results were obtained using a 25+ threshold to indicate being overweight). Psychiatric distress was indicated at each interview by scores of 2 or more on the 12-item General Health Questionnaire (GHQ; scored using caseness method) [30], a well-validated scale for assessing psychiatric morbidity. Since one of the items on this scale refers to losing sleep over worry, this item was removed from the scoring, though analyses with the full scale produced similar results.

### Analysis

Analyses were conducted in Mplus 7 [31] and SPSS v21. Considering attrition, the analysis sample was weighted to the living baseline sample throughout [32]. Within the analysis sample, rates of missing information across the five waves for insomnia symptoms and most covariates, except GHQ, were in the range 7.8-13.8% (see Table 1). There was a particularly high rate of missing information for psychiatric distress (23.9%) as a questionnaire error at wave 3 meant the GHQ could not be scored correctly for respondents in the regional sample (though this kind of missingness can be assumed to be random). Multiple imputation (25 datasets) was used throughout in addition to attrition weighting [33, 34]. Imputations employed an unrestricted two-level model of the analysis variables (repeated measurements nested within persons; gender, occupational class, type 2 diabetes and attrition weights were included at the person level; insomnia symptoms, smoking, physical inactivity, poor diet, obesity, heavy drinking and psychiatric distress were included at the repeated measurement level). Multiple imputation performs efficiently for dealing with missing data in marginal structural models [35].

**Table 1:** Descriptive data

*N* = 996	Observed			Imputed & Weighted for Attrition	
	*N*	%	% missing	*N*	%
Person level (*n* = 996)					
Female	541	54.3	0.0	553	55.5
Manual Occupation	303	30.7	0.9	338	33.9
Type 2 Diabetes at wave 5	68	6.8	0.0	70	7.0
Repeated Measurement level (n = 4980)					
Insomnia	1873	43.6	13.8	2246	45.1
Current Smoking	1496	32.6	7.9	1718	34.5
Physical Inactivity	2315	50.8	8.5	2575	51.7
Poor Diet	2564	57.3	10.2	2859	57.4
Obesity	865	19.3	9.9	946	19.0
Heavy Drinking	808	17.6	7.8	852	17.1
Psychiatric Distress	1033	27.3	23.9	1399	28.1

After attrition-weighting and imputation, we performed a traditional logistic regression of type 2 diabetes on a measure of cumulative insomnia exposure (i.e. the total number of study waves in which insomnia symptoms were reported, not necessarily consecutively; range 0-5), conditioning on all covariates (including measures from all waves). ORs associated with this cumulative measure of insomnia exposure represent the additional risk for type 2 diabetes associated with each additional wave where insomnia symptoms were reported, approximating a dose-response relationship. We then compared results from this traditional analysis with estimates of the effect of cumulative insomnia exposure on type 2 diabetes from MSMs, i.e. using weights to adjust for covariates, rather than conditioning on them. For both methods, *p*-values <0.05 were considered significant.

Weights for MSMs were calculated within each imputed data set by estimating two sets of probabilities for observed exposure levels (numerator and denominator probabilities). Probabilities from each wave were then multiplied together, and the product of the numerator probabilities was divided by that for the denominator probabilities [20]. Probabilities for observed exposure levels were calculated from logistic regression models predicting insomnia at each time point (in SPSS v21; see Additional file 1 for more details), Numerator probabilities were estimated based on past exposure histories only, while denominator probabilities were estimated based on past exposure and covariate histories. Two sets of analysis weights were produced, one with and one without concurrent covariates included in the denominator model. Gender and social class were included in both models, thus assuming that these are confounders, rather than mediators of insomnia’s effect.

Including past exposure histories in the numerator and denominator produces stabilised weights, which, in comparison to simply taking the inverse of the probability of exposure, helps to avoid excessive variability in the weights [20]. We additionally tried truncating weights at approximately their 5 and 95 percentiles to ensure that results were not being overly driven by cases with extreme weights [36], but this did not affect the findings.

Before employing the weights in MSMs we checked whether the mean weight was close to one [36] and whether they were balancing covariate histories across those with and without insomnia. Balance in covariate histories was assessed using standardised mean differences in covariates at each time point after wave-specific weights were applied (see Additional file 2 for details). Standardised differences less than 0.1 were considered negligible [37]. Initial assessment of covariate balance indicated that some differences in past covariates remained after weighting. This prompted some minor modification of the weighting model until satisfactory balance was achieved (e.g. adding some interactions to the denominator model; see Additional file 1 for details).

## Results

Table 1 shows descriptive statistics for the observed and the imputed data, with imputed data also weighted for attrition. Physical inactivity, poor diet and insomnia were the most common risk factors across the five surveys. Obesity and heavy drinking were the least common. Proportions with particular characteristics in the weighted and imputed data were similar to those in the observed data, with slightly more insomnia, smoking, females and manual households (suggesting respondents with these characteristics were more likely to drop out or fail to respond).

**Fig. 2:**
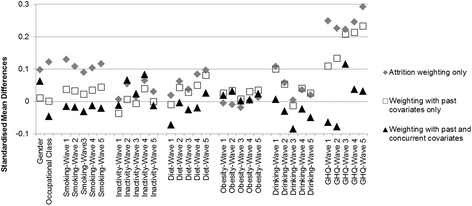
Standardised mean differences in covariates associated with cumulative insomnia exposure across 25 imputed data-sets

Next, we assessed the analysis weights. Means of the analysis weights with and without concurrent covariates were close to 1 at 1.026 and 1.007, respectively. Figure 2 shows standardised mean differences in covariates associated with cumulative insomnia exposure (i.e. with each additional wave where insomnia symptoms were reported; associations with insomnia at each wave using wave-specific weights are shown in Additional file 2). These are presented for attrition weighting only, and for the two sets of analysis weights with and without concurrent covariates included. Large mean differences in Fig. 2 (>0.1) suggest substantial co-variation with cumulative insomnia exposure. With attrition weighting only (grey diamonds) this co-variation may represent a mix of potential confounding and potential mediation (potential because a confounding or mediating role also depends on whether the covariate has a causal influence on type 2 diabetes). Thus, insomnia exposure shows substantial co-variation with gender, social class, smoking, baseline heavy drinking, and especially psychiatric distress. There was little co-variation with physical inactivity, obesity, later measures of drinking, or diet (though later measures of diet approached the 0.1 threshold).

With the analysis weights the potential confounding influences have been adjusted out, so any remaining co-variation is viewed as potential mediation. Covariates measured concurrently with insomnia could be potential confounders or potential mediators of insomnia exposure and the weights with and without these included respectively reflect these different causal assumptions (both sets of weights adjust for potential confounding from past measures of covariates). Thus, adjusting for confounding under the assumption that concurrent measures of covariates are potential mediators (‘weighting with past covariates only’; white squares in Fig. 2), removes co-variation with gender, occupational class and smoking, but not with baseline drinking or with psychiatric distress (though co-variation with psychiatric distress is attenuated). Adjusting for confounding under the more conservative assumption that concurrent measures of covariates are potential confounders (‘weighting with past and concurrent covariates’; black triangles in Fig. 2), removes associations with most covariates, though there is still a little co-variation with psychiatric distress, representing potential mediation (the association between cumulative insomnia exposure and GHQ at wave 3 is still >0.1).

Table 2 shows ORs, 95% CIs, and *p*-values for the association between cumulative insomnia exposure and type 2 diabetes incidence, as estimated with various methods. The basic association, weighted only for attrition, was that each additional wave of insomnia exposure was associated with increased risk for type 2 diabetes. A traditional regression model, conditioned on all the covariates, also showed this association (if anything it was stronger in the conditioned regression model). The association was attenuated but remained borderline significant in a MSM weighted for past covariates only, and all but disappeared in a MSM weighted for both past and concurrent covariates. In a sensitivity analysis with a less conservative threshold for diabetes (HbA_1c_ ≥ 6%), there was only a borderline association in models weighted for attrition only, and this was substantially attenuated in MSMs weighted for covariates (past only or with concurrent).

**Table 2:** Estimates of association between insomnia exposure and type 2 diabetes incidence

	Association between type 2 diabetes and cumulative insomnia exposure^a^		
Model	OR	95% CI	*p*-value
Attrition weighting only	1.26	1.03-1.54	0.028
Traditional logistic regression^b^	1.34	1.06-1.70	0.015
MSM weighted on past covariates only	1.20	0.98-1.46	0.079
MSM weighted based on past and concurrent covariates	1.08	0.85-1.37	0.554

^a^Results are weighted for attrition and averaged across 25 imputed datasets. Cumulative insomnia exposure is a count of study waves where insomnia symptoms were reported

^b^Conditioned on gender, baseline occupational class and smoking, physical inactivity, poor diet, obesity, heavy drinking, and psychiatric distress at each wave

## Discussion

Insomnia symptoms were investigated as a cause of type 2 diabetes incidence in a community-based sample of non-diabetic adults followed from approximately 36 to 57 years of age. MSMs were used to adjust for time-varying confounding. While other studies have shown associations with type 2 diabetes for shorter-term measures of insomnia [1, 2], this paper shows that risk for type 2 diabetes increases as exposure to insomnia symptoms accumulates over many years. However, association between insomnia symptoms and type 2 diabetes appeared largely confounded by other factors. Insomnia symptoms were also associated with being female, being in a manual class, smoking, heavy drinking and especially with psychiatric distress. The findings were sensitive to assumptions about the direction of causation between concurrent measures of insomnia symptoms and psychiatric distress (and to a lesser extent perhaps heavy drinking). Where concurrent measures of psychiatric distress were seen as potential mediators of insomnia effects, there was still a borderline association between insomnia symptoms and type 2 diabetes, which could represent a causal relationship, assuming no further unmeasured confounding. However, if concurrent measures of psychiatric distress were viewed as confounding insomnia effects, then the association with type 2 diabetes disappeared, suggesting a non-causal association.

The findings under the more conservative assumption of concurrent confounding contrast with small scale experimental studies where restricted sleep stimulates physiological changes similar to those in the development of diabetes [38, 39]. Experimental sleep restrictions are carefully controlled, short-term and may differ considerably from the experiences of those who, in non-experimental circumstances, report insomnia symptoms over the long-term (which is what has been studied here). Perhaps the physiological effects observed in short-term experimental studies do not persist when sleep is curtailed over the long-term. Alternatively, perhaps there is a (weak) causal effect of long-term insomnia exposure, assuming mediation via psychiatric distress. In addition, a study on the Penn State cohort showed highest risk for type 2 diabetes when insomnia symptoms were combined with short sleep duration [1]. Reporting insomnia symptoms does not necessarily equate with short sleep duration. Future studies might clarify whether long-term experience of insomnia with short sleep duration has a causal influence on type 2 diabetes.

The findings from MSMs also contrast with those from a meta-analysis of observational studies using traditional conditioned regression approaches [2]. MSMs have two main advantages over traditional regression in that they allow for mediated effects via time-varying covariates, and avoid potential collider biases [20, 22]. Assuming covariates increase risk for type 2 diabetes, allowing for mediated effects might be expected to yield less conservative effect estimates than with traditional regression, rather than *vice versa* as observed here. That the MSM estimates were more conservative therefore suggests the presence of an unmeasured factor that confounds the association between one of the covariates and type 2 diabetes, introducing collider bias when estimating effects of insomnia (see Fig. 1b). This could be nothing more than prior unmeasured histories of the time-varying confounders. Non-collapsibility of ORs may also account for some minor differences between conditioned and marginal effect estimates [40], but this is unlikely to have been an issue here as the outcome was rare.

In comparison to more traditional approaches, marginal structural models have been known to yield results closer to those of randomised experiments [41], but can be sensitive to mis-specification of the exposure model [36]. Hence, here we examined results using two sets of weights from different versions of this model, and performed further sensitivity analyses with different variable definitions. The findings were robust across these variations (if anything showing less evidence for a causal effect in some analyses), as well as to truncation of the weights to avoid extreme values dominating the analysis. However, the estimates of insomnia’s effects here do assume a linear, dose-response, relationship between insomnia exposure and type 2 diabetes, independent of other variables. Future studies might explore non-linearity of effects, or interactions with other variables such as psychiatric distress. For example, we modelled insomnia exposure as cumulative with greatest risk for type 2 diabetes among those experiencing chronic insomnia over all five interviews. Future work could examine whether effects of intermittent and chronic patterns differ more than would be expected by the differences in duration of exposure.

Effects estimated from marginal structural models, as for traditional regression, only indicate causation (or lack thereof), with the important assumption of no further unmeasured confounding [20]. Although adjustment was made for time-varying confounding within the 20-year period of study, some respondents may, for example, have had chronic patterns of insomnia symptoms that extended prior to the baseline measures, and any causal influence of earlier unmeasured insomnia symptoms on baseline covariates will have been adjusted out. Additionally, due to blood samples (for HbA_1c_) only being taken at the final wave of the study, we used an objective measure of type 2 diabetes from that final wave. Therefore, whilst we excluded those with baseline diabetes, based on self-report data, it is possible that early development of type 2 diabetes within the period of study (diagnosed or not) might have influenced the levels of time-varying exposures or covariates.

## Conclusions

Our findings were sensitive to method. Traditional logistic regression showed an association between insomnia symptoms and type 2 diabetes incidence which would be quite large in magnitude if multiplied by long-term exposure. However, this method may be subject to bias from time-varying confounders. Marginal structural models, which overcome these potential biases indicated considerable attenuation in the association, the extent of which depended on assumptions about causal direction between concurrently measured insomnia symptoms and covariates, particularly psychiatric distress. The attenuated association under those methods least likely to introduce bias, suggests that associations between insomnia symptoms and type 2 diabetes are largely confounded by other factors. Whilst this study is not the equivalent of a randomised trial, such trials can be expensive and lengthy, and it might be better to focus research resources on other potential modifiable causes where there is stronger observational evidence for a causal link. Psychiatric distress might be a good candidate for further investigation since it appeared to be the strongest potential confounder of the association between insomnia and type 2 diabetes incidence. Indeed, in this regard, treatment for insomnia may still have some value as this can help reduce psychiatric distress, and the strongest evidence here of insomnia being causally related to type 2 diabetes was when concurrent measures of psychiatric distress were viewed as mediators, rather than confounders.

## Additional files

Additional file 1: Appendix 1. Calculation of weights. Gives further technical details of the specific models used to calculate analysis weights. (DOCX 17 kb)
Additional file 2: Appendix 2. Covariate balance. Includes 5 supplementary figures showing how wave-specific weights achieve covariate balance on prior measures of covariates. (DOCX 164 kb)

## Abbreviations

BMI: Body mass index
CI: Confidence intervals
GHQ: General Health Questionnaire
MSMs: Marginal Structural Models
SEP: Socioeconomic Position
