# Supplementary material for: Insomnia symptoms as a cause of type 2 diabetes Incidence: a 20 year cohort study
Source: BMC Psychiatry. 2017 Mar 16;17:94. doi: 10.1186/s12888-017-1268-4 (PMC5356374; doi:10.1186/s12888-017-1268-4)
Supplement: Additional file 1: Appendix 1. — Calculation of weights. Gives further technical details of the specific models used to calculate analysis weights. (DOCX 17 kb) [file 12888_2017_1268_MOESM1_ESM.docx]

**Appendix 1 – Calculation of weights**

The numerator probabilities, p’_k_, were estimated using a model as follows: where k represents the time point (or survey wave), a_k_ represents the observed exposure level at time point k, and ã_k-1_ represents the observed exposure history up to time-point k-1, and a always resolves to 0 where there is a negative sub-script (e.g. if k=1 then a_k-2_=a_-1_=0):

*logit* p’_k_(a_k_=1|ã_k-1_)= β_0_+ β_1_k+ β_2_k^2^+ β_3_k^3^+ β_4_a_k-1_+ β_5_a_k-2_+ β_6_a_k-3_+ β_7_a_k-4_

Denominator probabilities, p_k_, were estimated using a similar model with additional terms (shown below): x’_k_ representing observed values of a set of time-varying covariates (current smoking, physical inactivity, poor diet, obesity, heavy drinking, and psychiatric distress) up to time-point k; x_k_ representing observed values for these time-varying covariates measured at time-point k; v_0_ representing observed baseline covariates (gender and occupational class); and with x resolving to 0 when it has a negative sub-script (e.g. x_-1_=0):

*logit* p_k_(a_k_=1|ã_k-1_, x’_k_, v_0_)= β_0_+ β_1_k+ β_2_k^2^+ β_3_k^3^+ β_4_a_k-1_+ β_5_a_k-2_+ β_6_a_k-3_+ β_7_a_k-4_+ β_8_x_k_+ β_9_x_k-1_+ β_10_x_k-2_+ β_11_v_0_

For the set of weights without concurrent covariates the β_8_x_k_ term was omitted from the denominator model. Numerator and denominator probabilities represented each individual’s probability of having their observed level of insomnia (e.g. p_k_ if they had insomnia, 1- p_k_ if they did not).

After examining covariate balance, some further modifications to the weighting model were made, adding selected interactions, and extending covariate histories for heavy drinking and psychiatric distress to include all waves (i.e. adding β_12_x_k-3_ and β_13_x_k-4_ for these covariates; inclusion of two previous waves appeared sufficient to remove confounding for the other covariates).

Thus, the full denominator model for the probability of insomnia at k, for example, was as follows (PA=physical inactivity; PD=psychiatric distress):

*logit* probability_k_= β_0_+ β_1_k+ β_2_k^2^+ β_3_k^3^+ β_4_Insomnia_k-1_+ β_5_Insomnia_k-2_+ β_6_Insomnia_k-3_+ β_7_Insomnia_k-4_+ β_8_Gender_k_+ β_9_Class_k_+ β_10_Smoking_k_+ β_11_Diet_k_+ β_12_PA_k_+ β_13_Obesity_k_+ β_14_Drinking_k_+ β_15_PD_k_+ β_16_Smoking_k-1_+ β_17_Diet_k-1_+ β_18_PA_k-1_+ β_19_Obesity_k-1_+ β_20_Drinking_k-1_+ β_21_PD_k-1_+ β_22_Smoking_k-2_+ β_23_Diet_k-2_+ β_24_PA_k-2_+ β_25_Obesity_k-2_+ β_26_Drinking_k-2_ + β_27_Drinking_k-3_ + β_28_Drinking_k-4_ + β_29_PD_k-2_+ β_30_PD_k-3_+ β_31_PD_k-4_+ β_31_Gender*k+ β_32_Gender*k^2^+ β_33_Gender*k^3^+ β_34_Class*k+ β_35_Class*k^2^+ β_36_Class*k^3^+ β_37_PD_k-1_*k+ β_38_PD_k-1_*k^2^+ β_39_PD_k-1_*k^3^+ β_40_Gender*Insomnia_k-1_+ β_41_Gender*Insomnia_k-2_+ β_42_Gender*Insomnia_k-3_+ β_43_Gender*Insomnia_k-4_+ β_44_Class*Insomnia_k-1_+ β_45_Class*Insomnia_k-2_+ β_46_Class*Insomnia_k-3_+ β_47_Class*Insomnia_k-4_+ β_48_PD_k-1_*Insomnia_k-1_+ β_49_PD_k-1_*Insomnia_k-2_+ β_50_Class*Smoking_k-1_+ β_51_Class*Diet_k-1_+ β_52_Class*PA_k-1_+ β_53_Class*Obesity_k-1_+ β_54_Class*Drinking_k-1_+ β_55_Class*PD_k-1_+ β_56_Gender*Smoking_k-1_+ β_57_Gender*Diet_k-1_+ β_58_Gender*PA_k-1_+ β_59_Gender*Obesity_k-1_+ β_60_Gender*Drinking_k-1_+ β_61_Gender*PD_k-1_+ β_62_PD_k-1_*Smoking_k-1_+ β_63_PD_k-1_*Diet_k-1_+ β_64_PD_k-1_*PA_k-1_+ β_65_PD_k-1_*Obesity_k-1_+ β_66_PD_k-1_*Drinking_k-1_
