# Supplementary material for: Insomnia symptoms as a cause of type 2 diabetes Incidence: a 20 year cohort study
Source: BMC Psychiatry. 2017 Mar 16;17:94. doi: 10.1186/s12888-017-1268-4 (PMC5356374; doi:10.1186/s12888-017-1268-4)
Supplement: Additional file 2: Appendix 2. — Covariate balance. Includes 5 supplementary figures showing how wave-specific weights achieve covariate balance on prior measures of covariates. (DOCX 164 kb) [file 12888_2017_1268_MOESM2_ESM.docx]

**Appendix 2 –covariate balance**

Covariate balance was assessed using wave-specific weights (calculated by dividing the numerator by the denominator probability for that wave). If the weights were working correctly, then those with concurrent covariates included should, for example, remove differences in covariates up to the wave at which insomnia is measured but not necessarily remove differences in covariates measured after that wave (i.e. weighting for insomnia at wave 3 should remove covariates differences at waves 1-3 but not necessarily at waves 4 and 5). Figures A1a-A1e show differences associated with insomnia at waves 1-5 respectively using the wave-specific weights. Weighting based on past covariates tended to reduce associations between insomnia symptoms and covariates from previous waves, but leave associations with concurrent or future measures of covariates largely unchanged. Weighting that utilised past and concurrent covariates additionally reduced the concurrent (and sometimes future) associations.


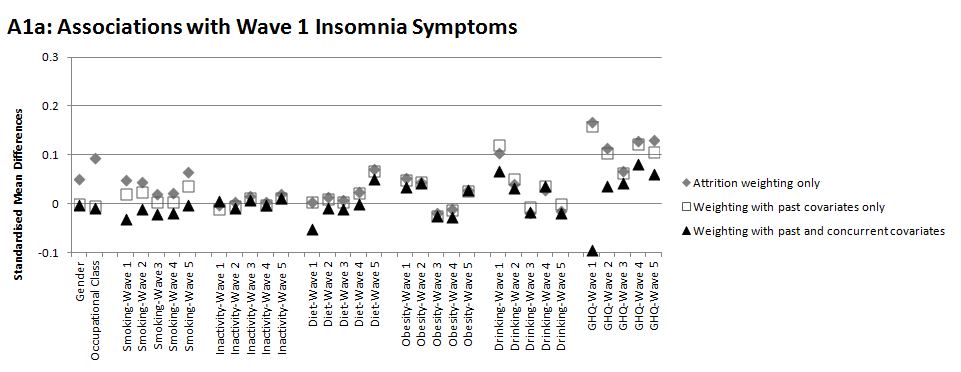


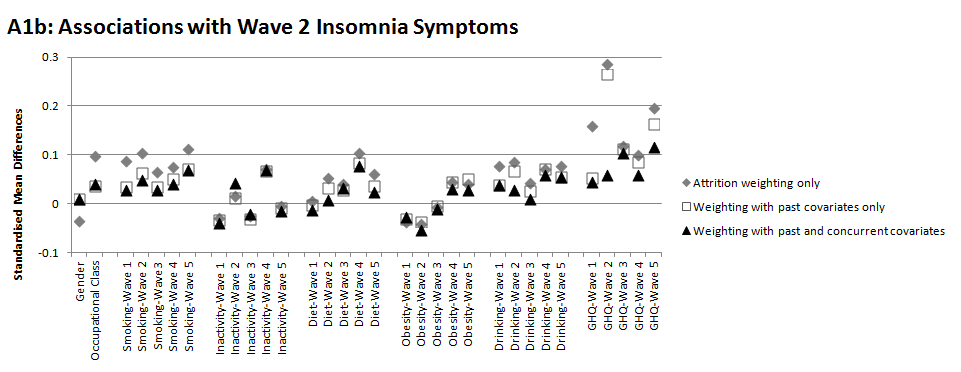


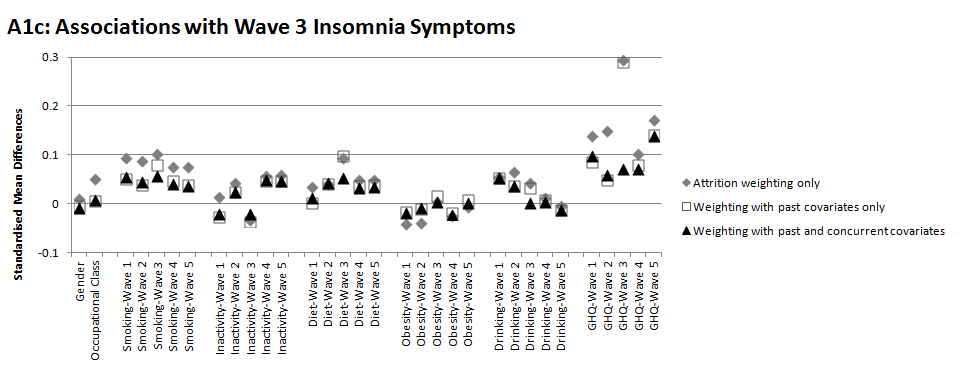


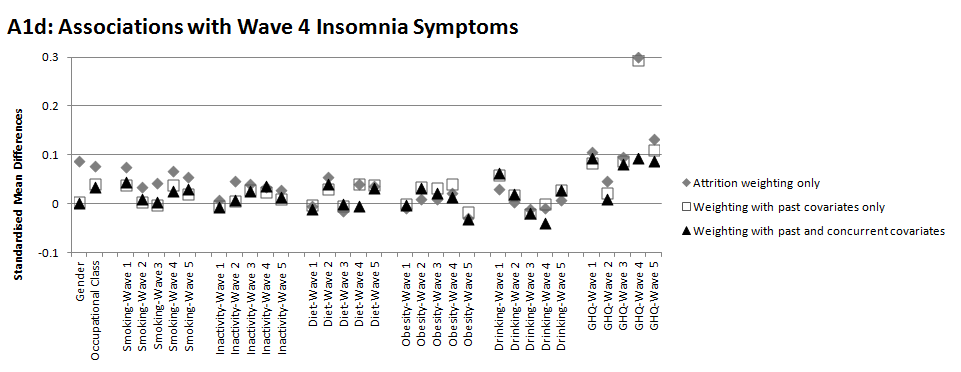


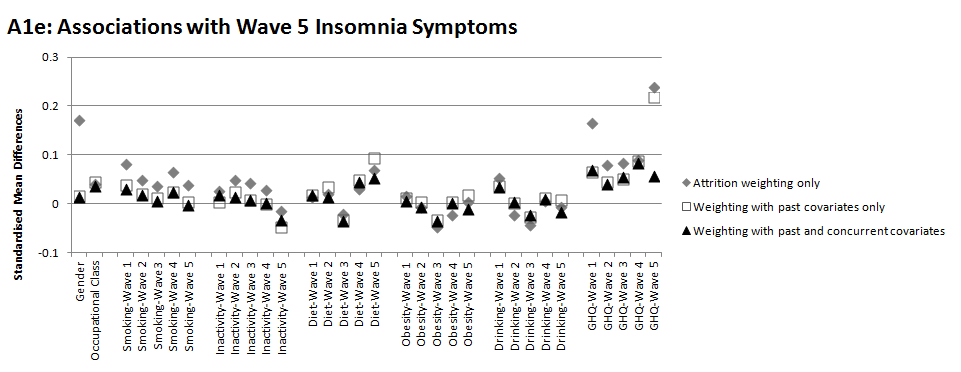


**Figure A1: Mean standardised differences in covariates associated with insomnia at specific waves**
